# miRNA and lncRNA as biomarkers in cholangiocarcinoma(CCA)

**DOI:** 10.18632/oncotarget.19044

**Published:** 2017-07-06

**Authors:** Bo Zheng, Seogsong Jeong, Yanjing Zhu, Lei Chen, Qiang Xia

**Affiliations:** ^1^ International Cooperation Laboratory on Signal Transduction, Eastern Hepatobiliary Surgery Institute, Second Military Medical University, Shanghai 200438, P.R. China; ^2^ National Center for Liver Cancer, Shanghai 201805, P.R. China; ^3^ Department of Liver Surgery, Renji Hospital, School of Medicine, Shanghai Jiao Tong University, Shanghai 200127, P.R. China

**Keywords:** miRNA, lncRNA, biomarker, cholangiocarcinoma, prediction

## Abstract

The microRNAs are a group of 20 nucleotides-long non-coding RNAs. By binding to the 3’UTR region of target mRNA, microRNAs can perform extensive actions mediating gene expression at post-trancriptional stages. It makes microRNAs serve as very crucial regulators in various biological progress including carcinogenesis. Long non-coding RNAs, however, are a subgroup of RNA with the length of 200 nucleotides. Unlike microRNAs, long non-coding RNAs can form secondary of tertiary domain based on their length. With the ability of directly interacting with DNA, RNA, protein, long non-coding RNAs have promoting or inhibitive functions in gene expression regulation. Furthermore, the abnormal expression of certain long non-coding RNAs has roused people’s interest in the role of long non-coding RNAs in tumorigenesis. Although the connection between microRNA/long non-coding RNA and CCA has been a hot field to researchers, the link between molecular mechanism and clinical outcome has been barely built. This review takes a retrospect at the latest researches on the link between microRNA/long non-coding RNA and cholangiocarcinoma and the potential of microRNA/long non-coding RNA serving as distinctive biomarkers for CCA in clinical practice.

## INTRODUCTION

Cholangiocarcinoma which can be anatomically divided into intrahepatic cholangiocarcinoma (ICC) and extrahepatic cholangiocarcinoma (ECC) accounts for 10% of primary hepatic tumors [1, 2]. Because of the highly molecular heterogeneity, CCA patients have been linked with bad prognosis and limited therapeutic regimens. Most of CCA are unresectable when discovered since it has progressed into advanced stages. So the improvement in the diagnostic method of CCA is urgent, especially in biomarkers. The classic serum CCA markers such as carbohydrate antigen CA-199 and CA-125 are now regarded as insensitive and unspecific. Since gene expression pattern is greatly changed in CCA background, it is of urgency to identify new epigenetic biomarkers such as miRNA and lncRNA for CCA patients [3].

**Figure 1:**
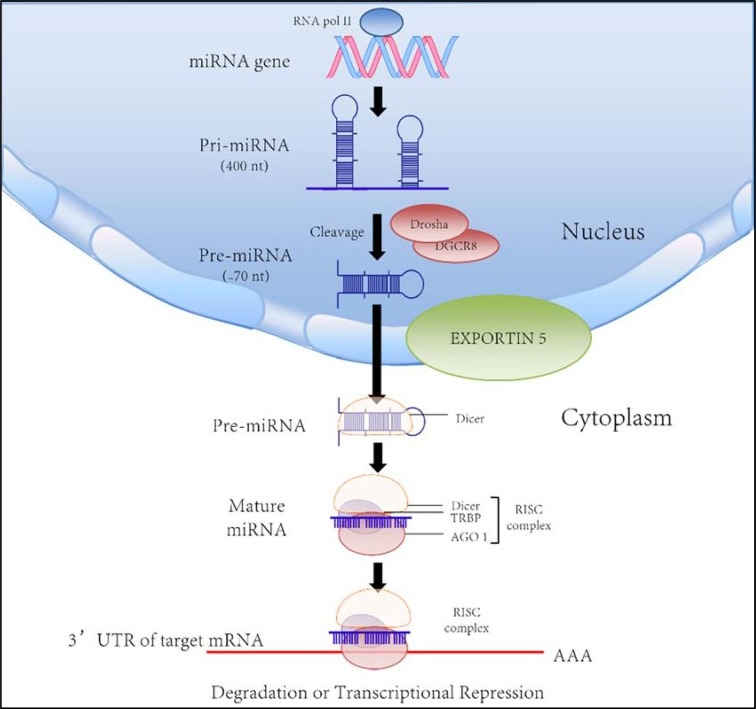
Biogenesis and biological function of microRNA [44]

MicroRNAs (miRNAs) refer to a subgroup of small, noncoding RNA that mediate a series of biological events from controlling the growth of insects to deactivating X chromosome in mammals [4, 5]. The first miRNA is found in the nematode *Caenorhabditis elegans,* but soon researchers discovered that there are thousands of them existing in nearly every speices [6, 7]. However, the exact mechanism of their function is not fully understood yet. Originally, miRNAs derive from long primary miRNAs with several hundred nucleotides in length [8–10]. After cleavage and processing, the small molecule goes through 60–70 nt long precursor and finally becomes 20–25 nt long miRNAs duplexes [11–17]. Then miRNAs will form into RNA-induced silencing complex (RISC). The complex will degrade target mRNA through complementary binding, therefore affecting translational activity [18–21] (Figure 1). Furthermore, a single miRNA has the potential of targeting over hundreds of mRNAs and each mRNA can be targeted by different miRNAs as well which makes the interactive network between miRNA and mRNA even more complex.

Although previous studies have confirmed that dysregulated miRNAs have a crucial role in tumors [22–26], the underlying mechanisms remain largely unknown. Some miRNAs like miR-21 [27, 28], miR-155 [29, 30] and miR17-92 cluster are recognized as tumor promoter [31, 32], some as tumor suppressors [33]. Although currently the therapeutic application of miRNAs in tumors is still limited, recently researchers have changed their focus to profiling miRNAs in tumor tissues so that miRNAs can be used as predictive, therapeutic or prognostic biomarkers in tumor patients. A research in 2006 first confirmed the biomarker role of miRNAs in CCA patients [34].

**Figure 2:**
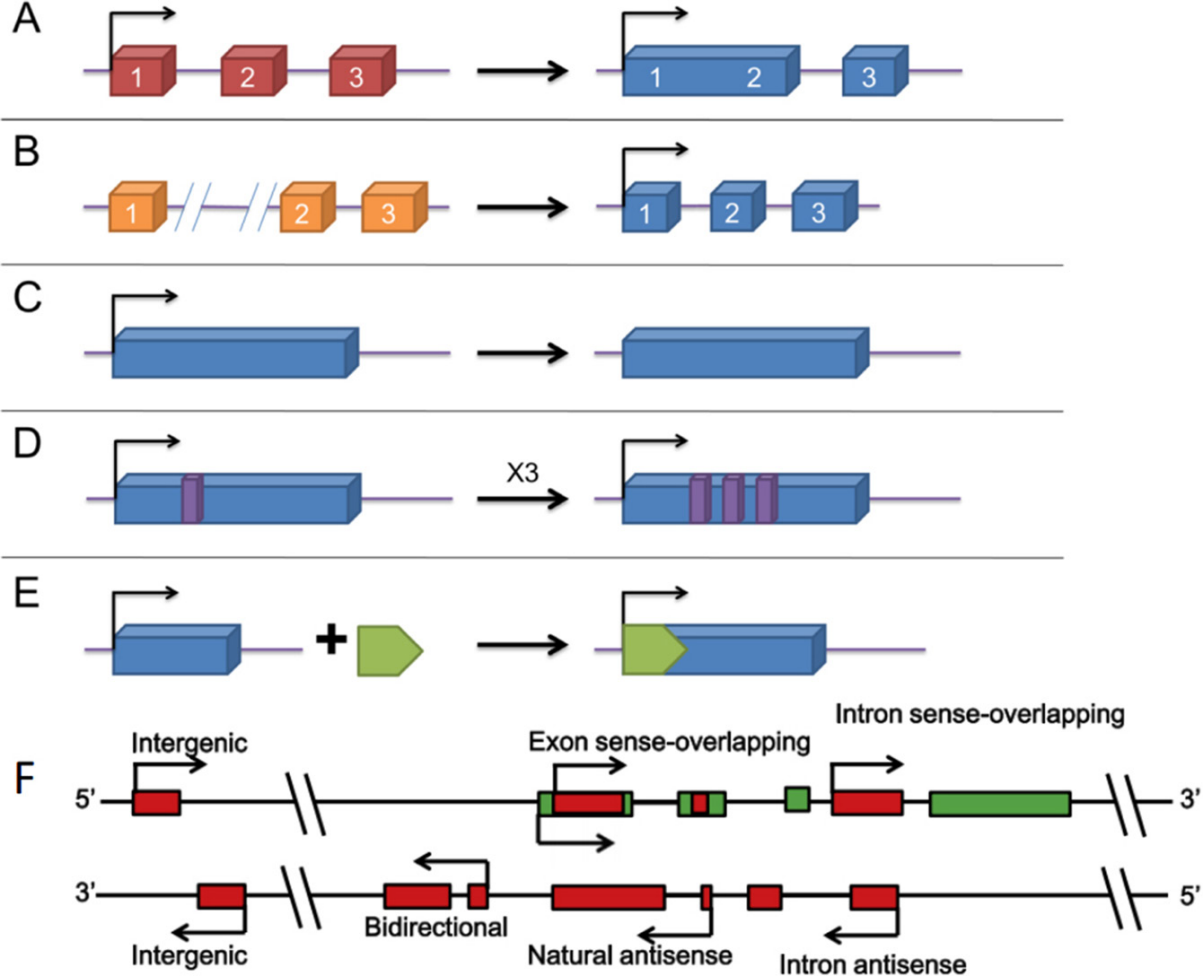
The origins of lncRNAs [87, 88] (**A**–**E**) different modes of lncRNA formation. (A) a lncRNA is transformed from a protein-coding gene with structural damage; (B) two abreast non-transcribed regions generate a lncRNA after chromosomal rearrangement; (C) copy of a noncoding gene by retrotransposition forms a lncRNA without protein-coding ability; (D) a lncRNA with adjacent repeats derives from tandem duplication events; (E) a functional lncRNA with insertion of a transposon. (**F**) Different kinds of lncRNA genes encode different lncRNAs. Intergenic: a lncRNA gene lies as an independent unit within the genomic interval between two genes. Bidirectional: expression of a lncRNA gene and its neighboring coding transcript on the opposite strand is initiated in close genomic proximity. Intron sense-overlapping: a lncRNA gene lies in the intron of a protein-coding gene on the same strand. Exon sense-overlapping: a lncRNA gene lies in the exons of protein-coding gene on the same strand. Intronic-antisense: a lncRNA lies in the introns of protein-coding gene on the opposite strand in the same region. Natural-antisense: a lncRNA gene lies in the exons of protein-coding gene on the opposite strand.

**Figure 3:**
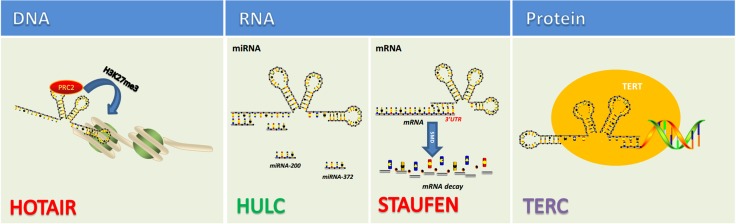
lncRNAs function via directly interacting with DNA, RNA, protein [89, 90]

When first discovered, long non-coding RNA (lncRNA) was once called transcriptional noise. However, more and more researches have demonstrated that lncRNAs affect various biological processes including tumorigenic process. Just like mRNA, lncRNAs are transcribed by RNA polymerase II and also have the structural feature of 5′-7-methylguanosine cap and 3′-poly(A) tail [35–38] (Figure 2). The localization and expression of lncRNAs differ in different tumors [39]. In fact, the localization of lncRNA remains ambiguous as some proven existing in nucleus or cytoplasm and some in both [40]. The broad existence of lncRNAs enables them to engage in a wide range of biological processes from chromatin remodeling to translational mediation. To achieve these, lncRNAs have to interact directly with DNA, protein, mRNAs and miRNAs (Figure 3). According to the RNA-seq result of 7 paired ICC and paracancerous tissue, the expression of 230 lncRNAs and 2220 mRNAs are dysregulated in tumor tissues compared with adjacent normal tissues [41]. Among them, 597 mRNAs were targeted by 169 lncRNAs with 219 negatively correlated and 550 positively correlated [41]. Recently, lncRNAs have been found functioning as the downstream molecule of oncogenes and tumor suppressor genes [42]. In this case, lncRNAs can be classified into ‘OncoLncRNA’ and ‘TSLncRNA’ [42]. In a transcriptomic profiling, researchers found that 2148 lncRNAs significantly upregulated, 568 downregulated in ICC tissues which shows big promises to identify certain lncRNAs as biomarkers in ICC patients [43].

### miRNA as biomarkers in CCA

Most of previous studies put their focus on the promoting or inhibitive role miRNAs might have in the tumorigenic process of malignant tumors such as CCA. However, more and more researchers have seen the potential of miRNAs serving as biomarkers of diagnosis, prediction, monitoring, therapy and prognosis for CCA patients. Cholangiocarcinoma hold the ability to release small molecules such as miRNAs into bloodstream or bile which further potentiate miRNAs to be the biomarker of CCA (Table 1).

**Table 1:** microRNAs as potential biomarkers for CCA

MicroRNA	Expression	Detectable location	Biomarker category	Sensitivity (%)	Specificity (%)	Target gene	Tumor type
191 [49]	Upregulated	Tissue/serum	Diagnostic/Prognostic	_	_	TET1/P53	ICC
29a [56, 107]	Upregulated	Tissue	Prognostic	_	_	HDAC4	ICC/ECC
21 [34, 57, 108]	Upregulated	Plasm	Diagnostic/Prognostic/Chemoresistance	87.8	90.5	PTEN, PTPN12, PTPN14	ICC
221 [57]	Upregulated	Plasm	Diagnostic	_	_	DDIT4	ICC
150-5p [62, 109]	Downregulated	Serum/bile/tissue	Diagnostic	93.3	53.5	ELK1	ICC/ECC
122 [66, 110]	Downregulated	Tissue	Prognostic	_	_	P53	ICC/ECC
200a [71, 111]	Downregulated	Tissue	Prognostic	_	_	rho-kinase 2/SUZ12	ICC/ECC combined with hepatolithiasis
203 [72]	Downregulated	Tissue	Prognostic	_	_	_	ICC/ECC
26a [73, 112]	Upregulated	Serum	Diagnostic/Prognostic	84.8	81.8	Keratin 19	CCA
29b/205/221 [75]	Downregulated	Tissue	Chemoresistance	_	_	PIK3R1/MMP-2(29b) ErbB3/ VEGFA(205) PIK3R1(221)	ICC/ECC
151-3p/126 [77]	Downregulated(151-3p) Upregulated (126)	Tissue	Prognostic	_	_	_	Resected ICC/ECC
590-3p [80]	Donwregulated	Serum	Diagnostic/Prognostic	_	_	SIP1	ICC
192/21 (combined) [84, 113]	Both upregulated	Urine	Diagnostic	63.6(192) 63.6(21) 81.8(combined)	66.7(192) 71.4(21) 71.4(combined)	PDCD4	O.viverrini-infected ICC/ECC
192 [85]	Upregulated	Serum	prognostic	74	72	_	O.viverrini-infected ICC/ECC
miRNA-483-5p,miRNA-505-3p, miRNA-874,miRNA-885-5p, miRNA-320b, miRNA-92b-3p, miRNA-1275,miRNA-1307-3p(panel)[86]	_	Plasma	Diagnostic	_	_	_	O.viverrini-infected ICC/ECC
373 [104]	Downregulated	Tissue	Prognostic	_	_	MBD2	ICC/ECC
miR-191, miR-486-3p, miR-1274b, miR-16 and miR-484(panel)[114]	_	Bile	Diagnostic	67	96	_	ICC/ECC
34a [115]	Downregulated	Tissue	Prognostic	_	_	Smad4	ECC
224 [116]	Upregulated	Tissue	Prognostic	_	_	IL-6	ICC/ECC
204 [117, 118]	Downregulated	Tissue	Prognostic	_	_	Slug	ICC/ECC

Abbreviations: TET1, ten-eleven translocation 1; HDAC4, Histone deacetylase 4;PTEN, phosphatase and tensin homolog;PTPN, protein tyrosine phosphatase;DDIT4, DNA-damage inducible transcript 4;ELK1, Ets including gene-1; PIK3R1, Phosphoinositide-3-kinase regulatory subunit 1;MMP-2, Matrix metalloproteinase 2;ErbB3, erythroblastic leukemia viral oncogene homolog 3;VEGFA, Vascular endothelial growth factor A;SIP1, Smad-interacting protein 1; PDCD4, programmed cell death 4;MBD2, Methyl CpG Binding Domain protein.

### miRNA-191

MiRNA-191 has been found upregulated in various solid malignancies [45–48]. A recent research firstly discovered that the expression of miRNA-191 is increased in ICC tissues and subsequently promotes the proliferation, infiltration and metastasis of tumor cells [49]. According to the research, the miRNA-191-TET1(ten-eleven translocation 1)-P53 axis is held responsible for the pro-tumor role of miRNA-191 in ICC [49]. In a cohort study, miRNA-191 is identified as an independent risk factor of disease-free survival and OS (overall survival) in ICC [49]. It is also found that the high expression of miRNA-191 in tumor tissue is linked with more advanced tumor stage and decreased OS which clearly implies miRNA-191 can be used as a tissue biomarker for predicting the OS of ICC patients [49]. In addition, by using the GEO database (GSE59856), researchers surprisingly discovered that the serum level of miRNA-191 is significantly higher in ICC patients (98) than healthy people (150) which may shed light on using miRNA-191 as a predictive serum biomarker in ICC in the future [49].

### miRNA-29a

The expression of miRNA-29a is found upregulated in myeloid leukemia, breast cancer, glioma and nasopharyneal carcinoma [50–52], while downregelated in pancreatic cancer, prostate cancer and gastric cancer [53–55]. The finding indicates that miRNA-29a might have a duel pro/anti-tumor function in human tumors. In CCA tissues, the expression of miRNA-29a is markedly increased [56]. Additionally the increased expression of miRNA-29a is closely associated with the differentiation, clinical stage, metatstasis of CCA [56]. By using statistic method as KaplanMeier survival analysis and Cox regression analysis, the overexpression of miRNA-29a is correlated with patients’ bad prognosis and miRNA-29a is considered as an independent risk factor in CCA [56]. Above all, the results indicate that miRNA-29a might become a prognostic tissue biomarker for CCA patients.

### miRNA-21/221

Through RNA sequencing, researchers found that the expression of miRNA-21, miRNA221 is highly increased in ICC [57]. Notably, both of them can be detected in the plasma of ICC patients with a higher concentration than healthy people, indicating their use as diagnostic biomarker in ICC patients [57]. The expression level of miRNA-21 in the plasma of ICC patients is as 3 fold much as healthy people, emphasizing its diagnostic role in ICC [57]. Moreover, the increased expression of miRNA-21 is correlated with more invasive and metastatic behavior in cholangiocarcinoma cells [58]. This finding may indicate that miRNA-21 can also become a novel prognostic plasma biomarker for ICC, however, large scale clinical studies need to be performed to support this hypothesis. Like miRNA-21, the expression of miRNA-221 is significantly increased in ICC patients. Previous studies have shown the pro-tumor function miRNA-221 had in hepatocellularcarcinoma, bladder cancer [59, 60]. Interestingly, a recent research found out the anti-tumor role of miRNA-221 in lung cancer [61].

### miRNA-150-5p

Microarray profiling results of normal tissue, PSC (primary sclerosing cholangitis, a major risk factor for CCA) and CCA show that the expression of miRNA-150-5p is significantly downregulated in CCA patients’ serum, bile and tissue [62]. Further experiments proved that the overexpression of miRNA-150-5p could block the proliferation and metastasis of tumor cells while the knockdown of miRNA-150-5p will lead to enhanced proliferation, invasion of tumor cells [62]. The expression of miRNA-150-5p is going through a dynamic change from normal tissue to PSC to CCA which means miRNA-150-5p may be able to inhibit the transition from chronic inflammation to CCA [62]. The increased level of CA19-9 is a clear diagnostic sign for ICC patients [63]. Current data indicates that there is a negative correlation existing between the expression level of miRNA-150-5p and the level of CA19-9 and the pathological stage of CCA [62]. According to this discovery, combined with CA19-9, miRNA-150-5p might serve as a predictive serum/bile/tissue biomarker for CCA in early detection and improve the overall prognosis for CCA patients.

### miRNA-122

MiRNA-122 is the most abundant miRNA expressed in liver cells. Its function includes mediating lipid metabolism, hepatitis C virus replication, cell differentiation and hepatic metabolism [64]. The decreased expression of miRNA-122 can lead to dysregulated liver function by disrupting normal mitochondrial functions. It is found that miRNA-122 deficiency may be closely associated with bad prognosis of CCA patients [65]. Coincidentally, the expression of miRNA-122 is markedly lower in cholangiocarcinoma tissues than normal bile duct tissues [66]. Taken together, miRNA-122 may serve as a promising prognostic tissue biomarker for CCA patients in the future.

### miRNA-200a

The expression of miRNA-200a is downregulated in a wide range of malignancies and further experiments demonstrated that miRNA-200a has an inhibitory role in many kinds of tumors [67]. Further researches confirmed miRNA-200a deficiency is correlated with the progression of certain types of cancers [67–70]. In ICC patients complicated with hepatolithiasis the expression of miRNA-200a is significantly downregulated in CCA tissues compared with normal bile duct tissues and simple hepatolithiasis tissues [71]. Moreover, the expression level of miRNA-200a is correlated with pathological stage and metastasis of CCA which makes miRNA-200a a potential prognostic tissue biomarker in CCA [71].

### miRNA-203

According to a previous study, the expression of miRNA-203 is significantly downregulated in CCA tissues compared with paracancerous tissue [72]. Combined with clinical data, further analysis shows that the expression level of miRNA-203 is closely associated with the size of tumor, differentiation of tumor cells and clinical stage [72]. Hence the miRNA-203 expression level might be a positive factor determining the prognosis of CCA patients [72]. Furthermore, the overall survival rate is much lower in miRNA-203 underexpression group than miRNA-203 overexpression group [72]. According to a multivariate Cox model, miRNA-203 is found to be an independent prognostic factor for CCA patients [72]. In conclusion, these findings suggest that miRNA-203 is a potential prognostic tissue biomarker for CCA.

### miRNA-26a

The expression level of miRNA-26a in serum is significantly upregulated in CCA patients than healthy people and closely associated with tumor stage [73]. In order to determine the potential of miRNA-26a in the early diagnosis of CCA, researchers compare the AUC curve of miRNA-26a with that of the classic biomarker CA19-9. Surprisingly, the AUC value of miRNA-26a is higher, suggesting the diagnostic power of miRNA-26a in the early diagnosis of CCA [73]. Furthermore, clinical data demonstrates that there is a clear correlation between miRNA-26a and adverse clinicopathological factors, progression-free and OS rate of CCA patients [73]. Through multivariate analysis, miRNA-26a is identified as an independent prognostic factor in CCA [73]. Taken together, miRNA-26a has the potential of serving as both diagnostic and prognostic serum biomarker in CCA patients.

### miRNA-29b/205/221

Gemcitabine (Gem; 2′,2′-difluorodeoxycytidine, dFdC) is a cytidine analogue and currently administered as a chemotherapy drug against cholangiocarcinoma [74]. MiRNA-29b and miRNA-221 are found downregulated in Gem-resistant CCA cell lines and ectopic increase of their expression can restore chemosensitivity of CCA cells towards Gemcitabine [75]. Furthermore, it is found that the overexpression of miRNA205 might grant chemosensitivity towards Gemcitabin to innately Gem-resistant cholangiocarcinoma cells [75]. Taken together, miRNA-29a/205/221 show promises serving as tissue biomarkers of chemosensitivity in CCA patients. Furthermore, trabectedin, an anti-metastatic agent in CCA has been found to inhibit metastasis via downregulating the expression of gene SYK and LGALS1 [76]. These two genes are the direct targets of certain miRNAs such as MiR-21-3p, miR-21-5p and miR-31-3p and miR-1207-5p, miR-1225-5p separately, suggesting the overexpression of these miRNAs is closely associated with the effectiveness of trabectedin [76].

### miRNA-151-3p/126

Resection is the only curative treatment for CCA but the post-surgery survival rate is not promising. By analyzing the RNA extracted from resected CCA tissue, 43 miRNA were found significantly dysregulated in tumor tissue than adjacent normal tissue [77]. Of these dysregulated miRNAs, miRNA-151-3p and miRNA-126 were closely associated with post-surgery survival rate though the expression level of them are not correlated with the clinicalpathologic changes [77]. The upregulation of miRNA-151-3p and downregulation of miRNA-126 confer a better prognosis with median survival of 58.7 months than others with median survival of 15.1 months [77]. Above all, miRNA-151-3p and miRNA-126 might serve as a prognostic tissue biomarker for CCA patients who had undergone tumor resection.

### miRNA-590-3p

miRNA-590-3p has been found downregulated in various malignancies [78]. In bladder cancer cell lines, the upregulation of miRNA-590-3P has been associated with increased tumor invasiveness [79]. A recent study revealed that miRNA-590-3p is also downregulated in serum, tissue, cell lines of ICC, especially in those with metastatic potential [80]. Further researches confirmed that miRNA-590-3p ameliorated the process of epithelial-to-mesenchymal transition (EMT) by targeting EMT-activator Smad-interacting protein 1(SIP1) [80]. By using AUC curves and Cox proportional hazards mode, the serum level of miRNA-590-3p is identified as an independent diagnostic and prognostic serum biomarker for ICC patients [80]. Intriguingly in another study, miRNA-590-3p is found upregulated in hepatocellular carcinoma (HCC), accelerating tumorigenic process via targeting tumor suppressor gene PDCD4 and PTEN [81].

### miRNA and Opisthorchis viverrini-associated ICC

The incidence of ICC is higher in Southeast Asia than any other places in the world, where chronic liver fluke Opisthorchis viverrini (OV) infection has a major contributing role in the disease [82]. Moreover, the miRNA signatures are found unique in the subtypes of ICC with tumorigenic infection of OV [83].

In urine sample, miRNA-192 and miRNA-21 were found significantly higher in O. viverrini-infected patients with periductal fibrosis (PDF) and CCA than healthy people [84]. However, the expression level of miRNA-21 in urine is upregulated in patients with other inflammatory diseases as well [84]. Hence serving as a urine biomarker, miRNA-21 might not be specific enough. For this reason, the detection of miRNA-192 and miRNA-21 are combined together to enhance their diagnostic power. The result shows that the combination markedly increased the sensitivity and specificity in differentiation between O. viverrini-infected patients with PDF and CCA and healthy people [84]. The level of miRNA-192 is found higher in serum samples of OV-associated CCA as well [85]. The high level of miRNA-192 expression is positively correlated with lymph node metastasis and shorter OS [85].

In a 2015 research, OV-induced ICC tumor tissues, distal non-tumor tissues and normal non-tumor tissues from 14 CCA patients and normal tissues from healthy donors were analysed for miRNA expression level by using small RNA-Seq. The profiling result was then confirmed by performing quantitative PCR on paired plasma samples. 67 miRNAs are dysregulated with 35 upregulated and 32 downregulated in ICC tissues compared with distal non-tumor tissues and the dysregulated miRNA number reaches 316 with 144 upregulated and 172 downregulated when compared with normal non-tumor tissues [86]. Since OV-induced ICC shows distinctive challenges in early diagnosis and prognostic prediction, applicable biomarkers for early diagnosis in blood is urgently needed. Interestingly, out of all dysregulated miRNAs 8 miRNAs (miRNA-483-5p, miRNA-505-3p, miRNA-874, miRNA-885-5p, miRNA-320b, miRNA-92b-3p, miRNA-1275, miRNA-1307-3p) are found only detectable in the plasma of ICC patients [86]. This 8 miRNA panel might serve as a novel circulating miRNA-based biomarker for OV-induced ICC patients.

### lncRNA as biomarkers in CCA

LncRNAs have been found holding multiple biological functions ranging from cellular proliferation to cellular apoptosis [91]. Moreover, a close connection between dysregulated lncRNA expression and the prognosis of tumor patients has been discovered [92].

### LncRNA AFAP1-AS1

LncRNA AFAP1-AS1 is transcribed from the antisense DNA strand of gene AFAP1. Previous studies have demonstrated that AFAP1-AS1 is correlated with malignant behavior such as invasion and metastasis in hepatocellular carcinoma and nasopharyngeal carcinoma [93–95]. A recent study first discovered that the expression level of AFAP1-AS1 is significantly higher in CCA tissue than in paracancerous tissue and the same result was detected in CCA cell lines Hucct and normal biliary tract cells [96]. Further experiments showed that the underexpression of AFAP1-AS1 would restrict the proliferative and metastatic potential of CCA cell lines, Hucct and TFK-1 which confirmed that AFAP1-AS1 has a oncogenic function in cholangiocarcinoma [96]. Taken together, lncRNA AFAP1-AS1 might become a promising diagnostic and prognostic tissue biomarker for CCA.

### LncRNA CCAT1

LncRNA CCAT1(colon cancer associated transcript 1) was firstly found overexpressed in colorectal cancer, accelerating the tumorigenic process [97]. It is also demonstrated that the overexpression of CCAT1 is correlated with the occurrence, progression, chemoresistance of tumor cells [98–100]. A recent study showed that the expression level of CCAT1 is markedly higher in cholangiocarcinoma tissue than in paracancerous normal tissue [101]. Furthermore, the upregulation of CCAT1 might lead to bad pathological characteristics, lymph node metastasis and more advanced tumor stage [101]. Statistical data shows that the OS rate of lncRNA CCAT1-overexpressed patients is significantly lower than underexpressed patients and by using multivariate and ROC analysis, lncRNA CCAT1 is found to be an independent prognostic factor for CCA [101]. Taken together, lncRNA CCAT1 can serve as a prognostic tissue biomarker in CCA patients.

### LncRNA NEAT-1

BRCA-1 associated protein-1(BAP1) is identified as an anti-tumor factor and found participating in various cellular processes by interacting with other proteins. However, low BAP1 expressing CCA cell lines exhibit higher sensitivity to gemcitabine and cisplatin with lower IC50 [102]. Further studies demonstrated that lncRNA NEAT-1 is a down-stream molecule of BAP1 in treatment response [102]. In NEAT-1 knock-down CCA cell lines, the cytotoxicity of gemcitabine is significantly higher than control group which implies lncRNA NEAT-1 might serve as a chemosensitivity tissue biomarker for CCA.

### LncRNA MALAT1

The expression of lncRNA MALAT1 is upregulated in various kinds of cancers such as liver, uterus, lung, breast, prostate, pancreas and cervix [103]. Further studies confirmed that MALAT1 is an independent prognostic factor in some cancers as well [103]. In CCA especially hilar cholangiocarcinoma (HCCA), the expression level of MALAT1 is much higher than paracancerous tissue [104]. *In vitro* and vivo experiments, MALAT1 exhibits a pro-tumor function in the proliferation, invasion and migration of CCA cells [104]. Furthermore, overexpression of MALAT1 is correlated with lower OS rate, worse TNM stage, larger tumor size and metastasis in HCCA patients [104]. Taken together, lncRNA MALAT1 is a very promising novel prognostic tissue biomarker in HCCA.

### LncRNA CPS1-IT1

A 2015 study for the first time discovered that Carbamoyl-phosphate synthase 1 (CPS1) and its lncRNA CPS1 intronic transcript 1 (CPS1-IT1) are co-upregulated at the same time in ICC tissue compared with paracancerous normal tissue [105]. The study suggested that the overexpression of CPS1 and CPS1-IT1 is correlated with increased CA19-9 positivity and lymph node metastasis [105]. Further experiment confirmed that the upregulation of CPS1 and CPS1-IT1 has a negative impact on the OS rate for ICC patients [105]. In conclusion, current study suggests that CPS1-IT1 might serve as a prognostic tissue biomarker for ICC patients.

## CONCLUSIONS AND PROSPECTIVE

CCA has a bad prognosis and is still ranked as one of the most lethal malignant diseases. In recent years, many studies have uncovered the connections between miRNA/lncRNA and the cholangiocarcinoma in tumorigenic process and tumor progression, some of which suggested that miRNAs and lncRNAs might serve as novel biomarkers for CCA patients. Although in some studies, the sensitivity and specificity of these novel biomarkers are even better than the most widely used CA19-9, the clinical data supporting these findings are not big enough to be convincing. So in the next step of developing novel biomarkers, researchers should increase the number of their clinical candidates to make their discovery more statistically convincing. In conclusion, in the future miRNAs and lncRNAs may become very promising biomarkers for CCA in early diagnosis, treatment response and prognosis prediction.
